# Long term outcomes for elderly patients after emergency intensive care admission: A cohort study

**DOI:** 10.1371/journal.pone.0241244

**Published:** 2020-10-29

**Authors:** Ged Dempsey, Dan Hungerford, Phil McHale, Lauren McGarey, Edward Benison, Ben Morton

**Affiliations:** 1 Liverpool University Hospitals NHS Foundation Trust, Liverpool, United Kingdom; 2 Institute of Infection and Global Health, University of Liverpool, Liverpool, United Kingdom; 3 Department of Public Health and Policy, Institute of Population Health Sciences, University of Liverpool, Liverpool, United Kingdom; 4 Liverpool School of Tropical Medicine, Liverpool, United Kingdom; 5 Malawi Liverpool Wellcome Trust Clinical Research Programme, Blantyre, Malawi; Technion - Israel Institute of Technology, ISRAEL

## Abstract

**Background:**

Elderly patients (≥ 80 years of age) surviving an episode of critical illness suffer long-term morbidity and risk of mortality. Identifying high risk groups could assist in informing discussions with patients and families.

**Aim:**

To determine factors associated with long-term survival following ICU admission.

**Design:**

A cohort study of patients aged ≥ 80 years of age admitted to the ICU as an emergency.

**Methods:**

Patients admitted from January 2010 to December 2018 were included in the study. Primary outcome was five year survival. Mortality was assessed using a multivariable flexible parametric survival analysis adjusted for demographics, and clinically relevant covariates.

**Results:**

There were 828 patients. Mean age was 84 years (SD 3.2) and 419 (51%) were male. Patients were categorised into medical (423 (51%)) and surgical (405 (49%)) admissions. Adjusted hazard ratios (aHR) for mortality were highest for serum lactate (>8 mmol/l aHR 2.56 (C.I. 1.79–3.67)), lowest systolic blood pressure (< 70 mmHg aHR 2.04 (C.I. 1.36–3.05)) and pH (< 7.05 aHR 4.70 (C.I 2.67–8.21)). There were no survivors beyond one year with severe abnormalities of pH and lactate (< 7.05 and > 8 mmol/l respectively). Relative survival for medical patients was below that expected for the general population for the duration of the study.

**Conclusion:**

Overall five-year survival was 27%. For medical and surgical patients it was 19% and 35% respectively. Survival at 30 days and one year was 61% and 46%. The presence of features of circulatory shock predicted poor short and long term survival.

## Introduction

Almost 5% of the UK population were aged 80 years or more in the 2011 national census [1], a figure widely expected to rise in coming decades. This group is overrepresented in health care populations and comprises 10–20% of intensive care admissions [2–4]. However, despite improving outcomes [5], the benefit of intensive care interventions for many such patients are unclear, with conflicting outcome data in the literature [4, 6–8]. Post-critical care functional [9], cognitive and psychological morbidities are more pronounced in the elderly [10], with critical illness frequently exacerbating pre-existing frailty and/or functional impairment. Up to 75% of patients achieve a Palliative Performance Scale [11] score of less than 60 one year after intensive care unit (ICU) admission—a level of function associated with marked limitations of ambulation and a need for considerable assistance with self-care [9].

A prognostic scoring system that identifies those at highest risk of poor outcome beyond ICU discharge could assist in informing discussions with patients and families to establish their wishes regarding future health care interventions prior to ICU discharge.

Twelve-month survival rates are reported between 40 and 68% for very elderly patients (≥ 80 years of age) after ICU admission [12]. Studies consistently demonstrate that elective surgical patients have better outcomes than emergency and medical admissions [13–15]. The phenomenon of disproportionately increased usage of healthcare within the last 12 months of life is increasingly recognised [16]. Recent reports have suggested that elderly patients may not desire a “survival at all costs” approach [17, 18]. Forty three per cent of elderly interviewees indicated they would decline a period of invasive mechanical ventilation and 63% stated they would refuse renal replacement therapy after a period of invasive ventilation—preferring a lower intensity of care with primary focus on quality of life [17].

We conducted a retrospective analysis of prospectively collected data over a nine-year period to review outcomes for all patients aged 80 years and over with an unplanned or emergency admission to a tertiary ICU. Our aim was to identify prognostic and demographic factors that predict both short and long-term outcomes, and to compare their outcomes with expected survival of age-matched UK populations.

## Methods

### Setting

The study was conducted in the ICU of the Aintree University Hospital (AUH), site of Liverpool University Hospitals NHS Foundation Trust, Liverpool, UK. This is a 23-bedded mixed medical and surgical unit that admits 1300–1400 patients per year. The hospital is situated in an urban area north of the city centre, has 789 beds and serves some of the most socioeconomically deprived areas of the UK. AUH is the regional referral centre for trauma, hepato-biliary surgery and head and neck surgery.

### Study population

We included patients aged 80 years and over admitted to the ICU as an emergency between 1^st^ January 2010 and 31^st^ December 2018. Mortality data for the cohort was last updated on 31^st^ October 2019. Patients who underwent elective surgery were excluded. For patients admitted more than once during the study period, only data pertaining to the first admission was incorporated within the analyses. Readmission data were collected and recorded as either being during the same or separate hospital admissions.

Socioeconomic deprivation was assessed using the 2015 Index of Multiple Deprivation (IMD) based on home postcode (zipcode) mapped to Lower Super Output Area (LSOA) [19]. The IMD is a UK Government assessment of local deprivation based on LSOA. LSOAs are geographic areas designed to report small area statistics (mean population size, n = 1500) in England and Wales. There are 32,844 LSOAs in England that are ranked for deprivation across seven domains; income, employment and health deprivation, disability, barriers to housing and services, crime, living environment and education and skill deprivation. Each LSOA is assigned a deprivation score (IMD score) based on the seven domains which is subsequently ranked and then amalgamated into deciles with decile one being the most deprived and decile ten the least.

### Data collection

Patient data was collected prospectively as part of the UK national ICU audit programme (Intensive Care National Audit and Research Centre—ICNARC). Admissions were categorised as medical or surgical and data pertaining to pre-morbid status was collected, from case note review, using the Functional Co-morbidity Score (FCS) as described previously [20]. Acute physiological derangement was assessed using the serum lactate concentration, pH, the ratio of arterial partial pressure of oxygen to fraction of inspired oxygen (P_a_O_2_:F_i_O_2_), systolic blood pressure (SBP), plasma pH, serum lactate, Glasgow Coma Score (GCS), platelet count, serum creatinine and bilirubin. Mortality data was collected via the hospital electronic information system (Medway Sigma, System C Healthcare Ltd)

### Ethical approval

NHS Health Research Authority approval was granted (IRAS ID:220258). As this was a non-interventional study consent was deemed unnecessary.

### Variables

The primary outcome variable was patient survival five years following ICU admission. Secondary outcomes were survival at 30 days and one year. We selected the following covariates *a priori* to determine potential predictors of mortality: age, gender, medical / surgical admission, ICU readmission (within the same hospital admission), FCS, IMD score, P_a_O_2_:F_i_O_2_ ratio, SBP, plasma pH, serum lactate, GCS, platelet count, serum creatinine and bilirubin. All of the above variables (with the exception of ICU readmission) are available at, or shortly after, ICU referral and could potentially be used to inform admission decisions. For the purposes of constructing prediction model figures plasma pH, serum lactate, SBP, FCS and GCS were analysed as ordinal categorical variables, classified according to Table 1.

**Table 1:** Categorical variables and groupings used in analyses.

Variable	Grouping	Number of patients n (%)
**Functional co-morbidity Score**	0	108 (13)
	1	235 (28)
	2	244 (29)
	3	136 (16)
	≥ 4	105 (13)
**Glasgow Coma Score**	> 8	755 (91)
	≤ 8	73 (9)
**Systolic blood pressure (mmHg)**	< 70	35 (4)
	70–79	81 (10)
	80–89	158 (19)
	≥ 90	553 (67)
**Serum lactate (mmol/L)**	<4	549 (75)
	4–6	86 (12)
	>6–8	43 (6)
	>8	50 (7)
**Plasma pH**	< 7.05	22 (3)
	7.05–7.14	39 (5)
	7.15–7.24	119 (16)
	7.25–7.34	279 (37)
	≥ 7.35	298 (39)

### Statistical analysis

#### Relative survival analysis

Data were analysed using Stata V15 (StataCorp, Stata Statistical Software: Release 15.1, College Station, Texas, USA). Categorical variables were compared using the Chi^2^ test. Continuous variables were tested for normality and appropriate statistical tests applied (Table 2).

**Table 2:** Patient characteristics.

Characteristic	Overall n = 828	Medical patients n = 423	Surgical patients n = 405	P value
Age in years (sd)	84 (3.2)	83 (2.8)	84 (3.5)	<0.001*
Male gender n (%)	419 (51)	239 (56)	180 (45)	0.001**
Patients readmitted during study n (%)	34 (4)	17 (4)	17 (4)	0.897**
Patients readmitted during same hospital admission n (%)	21 (2.5)	9 (2.1)	12 (3)	0.445**
Mean APACHE II score (sd)	19 (6.0)	21 (6.3)	17 (5)	<0.001*
ICU deaths n (%)	235 (28)	154 (36)	81 (20)	0.002**
Hospital deaths n (%)	320 (39)	190 (45)	130 (32)	<0.001**
Median ICU length of stay in days (interquartile range)	3 (2–6)	3 (2–6)	3 (2–7)	0.143*
Total functional co-morbidity score—median (interquartile range)	2 (1–3)	2 (1–3)	2 (1–3)	0.04*
Socio-economic deprivation according to IMD decile n (%)				0.265*
1	282 (34)	149 (35)	133 (33)	
2	98 (12)	50 (12)	48 (12)	
3	80 (10)	51 (12)	29 (7)	
4	46 (5)	24 (6)	22 (5)	
5	76 (9)	33 (8)	43 (11)	
6	74 (9)	37 (9)	37 (9)	
7	47 (6)	23 (6)	24 (5)	
8	44 (5)	21 (5)	23 (6)	
9	57 (7)	26 (6)	31 (8)	
10	23 (3)	8 (2)	15 (4)	

ICU—Intensive care unit

APACHE—Acute physiology age and chronic health evaluation

IMD—Index of multiple deprivation

* Wilcoxon rank sum test

** Chi^2^ test

For a given calendar year and population stratum (sex and age), relative survival was defined as the ratio of survival among the ICU patients to the expected survival for the general UK population. Expected survival was estimated using data from life-tables obtained from the Office for National Statistics for the UK population and compares to our cohort for age, sex and year [21]. Relative survival was estimated using the STATA “strs” command, with our cohort split into medical or surgical admissions [22].

#### Multivariable survival analysis and predicted survival

We developed an *a priori* analysis plan to interrogate the above variables at admission. Patients alive at the end of five years, or at last follow up if less than five years, were censored. The relationship between these variables and five-year survival was initially explored with Cox-regression analysis. However, multiple variables (gender, SBP, P_a_O_2_/F_i_O_2_ ratio, lowest pH and lactate) were found to violate proportional hazard assumptions (S1 Table). Therefore, a flexible parametric survival model was fitted using the STATA “stpm2” command [23] to produce hazard ratios and related 95% confidence intervals (95% CIs), two-sided P-values of <0.05 were considered to indicate significance. The most parsimonious model which avoided overfitting was identified at five internal splines using the Bayesian information criterion method (S2 Table). Estimates from the flexible parametric model were similar to that of a Cox regression model (<5% difference in coefficients) [24]. Nomograms for possible prognostics factors associated with survival were therefore produced from the output from the Cox-regression model using R V.3.5.1 (R Development Core Team, Vienna, Austria).

## Results

**Fig 1:**
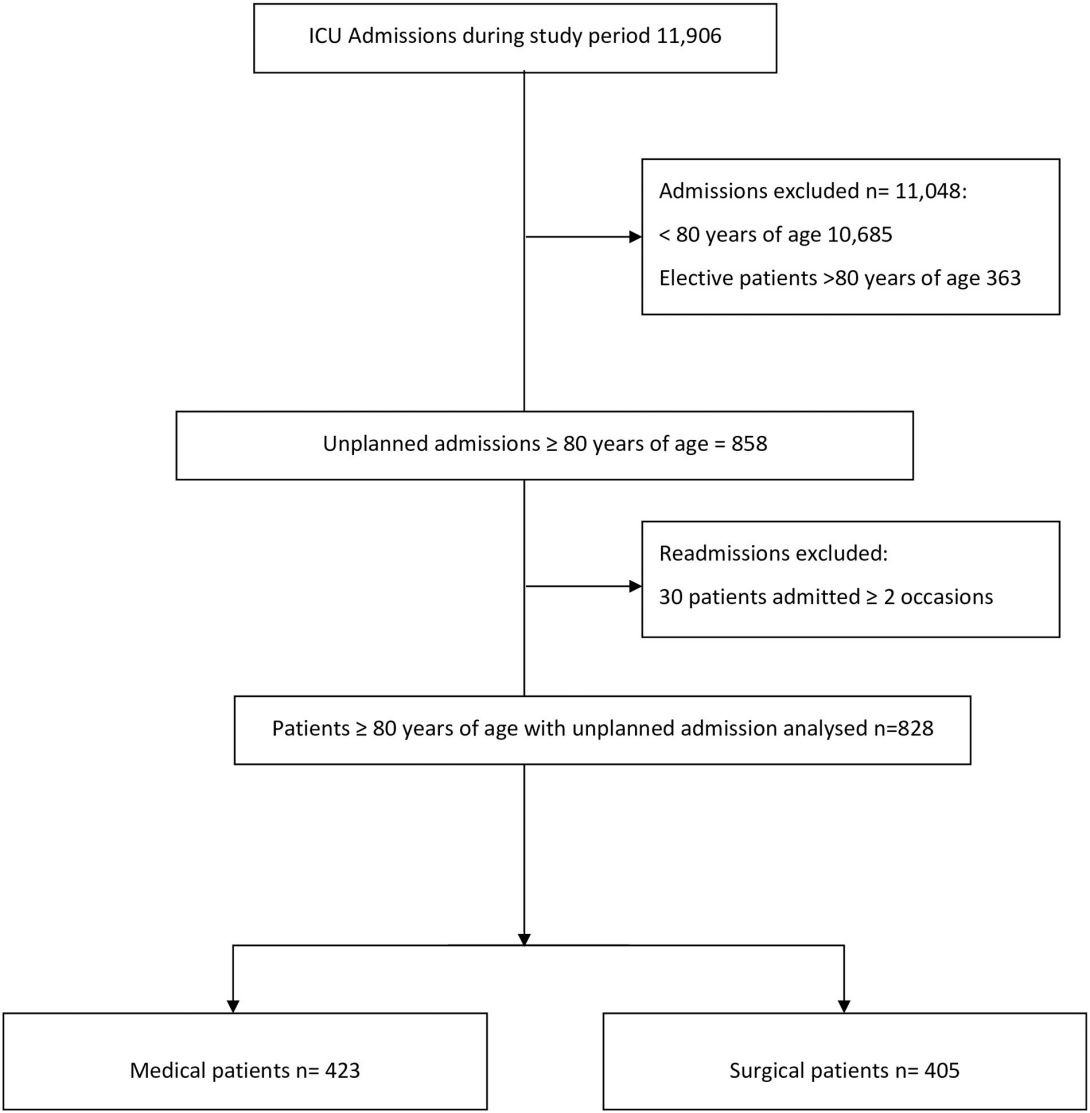
Patients aged 80 years and over admitted to the ICU during the study period.

During the study period there were 11,906 admissions to AUH ICU inclusive of 1221 (10.3%) patients aged ≥ 80 years. Of these, 858 (7.0%) were unplanned admissions. Thirty patients were admitted to the unit on ≥ two occasions leaving 828 patients for analysis (Fig 1) [25]. There were 423 (51%) medical patients and 405 (49%) surgical. Mean age of all patients was 84, 51% of all admissions were male (significantly higher for medical admissions, p = 0.001 χ^2^ Table 2), APACHE II score was significantly higher for medical than surgical admissions (21 versus 17, p<0.001 Wilcoxon rank sum Table 1), as was FCS (p = 0.04 Wilcoxon rank sum Table 2), percentage of ICU and hospital deaths (36% versus 20% and 45% versus 32% respectively, p<0.001 χ^2^ Table 2). There were no differences between the groups with respect to length of hospital stay (p = 0.143 Wilcoxon rank sum Table 2) and socioeconomic deprivation (p = 0.265 Wilcoxon rank sum Table 2). There was marked socioeconomic deprivation within the cohort, 34% of patients were from the most and only 3% from the least socioeconomically deprived deciles (Table 2).

### Relative survival analysis

**Fig 2:**
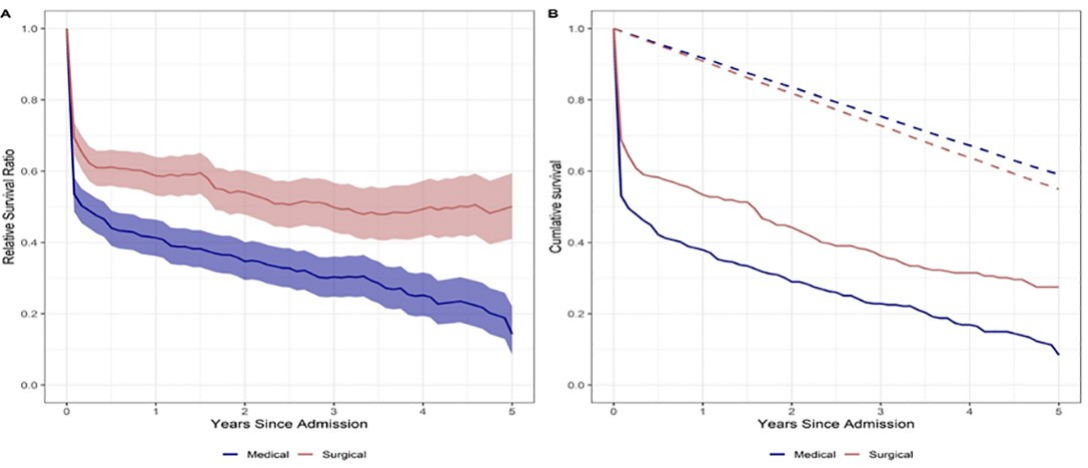
Modelled five-year survival of medical and surgical patients from ICU admission. Panel A illustrates the modelled relative survival over time. Panel B demonstrates modelled cumulative survival using the Kaplan Meier method comparing medical and surgical patients. In panel A the shaded area represents the 95% confidence intervals for the modelled relative survival ratio. In panel B the dashed lines represents the modelled cumulative survival of the UK reference population in relation to medical patients (blue dash) and surgical patients (red dash).

Survival curves are presented comparing medical and surgical patients to simulated UK average (Fig 2) and local deprived populations (S1 Fig). Survival at 30 days, one year and five years was 61%, 46% and 27% respectively, with survival for medical patients worse than surgical (S3 Table). Survival analyses were performed for patients alive up to five years from ICU admission and compared to non-critically ill populations. Relative survival analysis demonstrates that survival of surgical patients stabilised at approximately two years after ICU admission. Relative survival was 0.694 (95% CI 0.646–0.737) at 30 days, 0.587 (95% CI 0.532–0.640) at one year and 0.501 (95% CI 0.411–0.595) at five years. However, survival for medical patients continued to worsen compared to the non-ICU population for the duration of the study period (relative survival at 30 days, one and five years was 0.536 (95% CI 0.487–0.583), 0.414 (95% CI 0.363–0.464) and 0.143 (95% CI 0.085–0.220), respectively (Fig 2 and S3 Table).

### Clinical factors associated with survival

#### Univariable survival analysis

**Fig 3:**
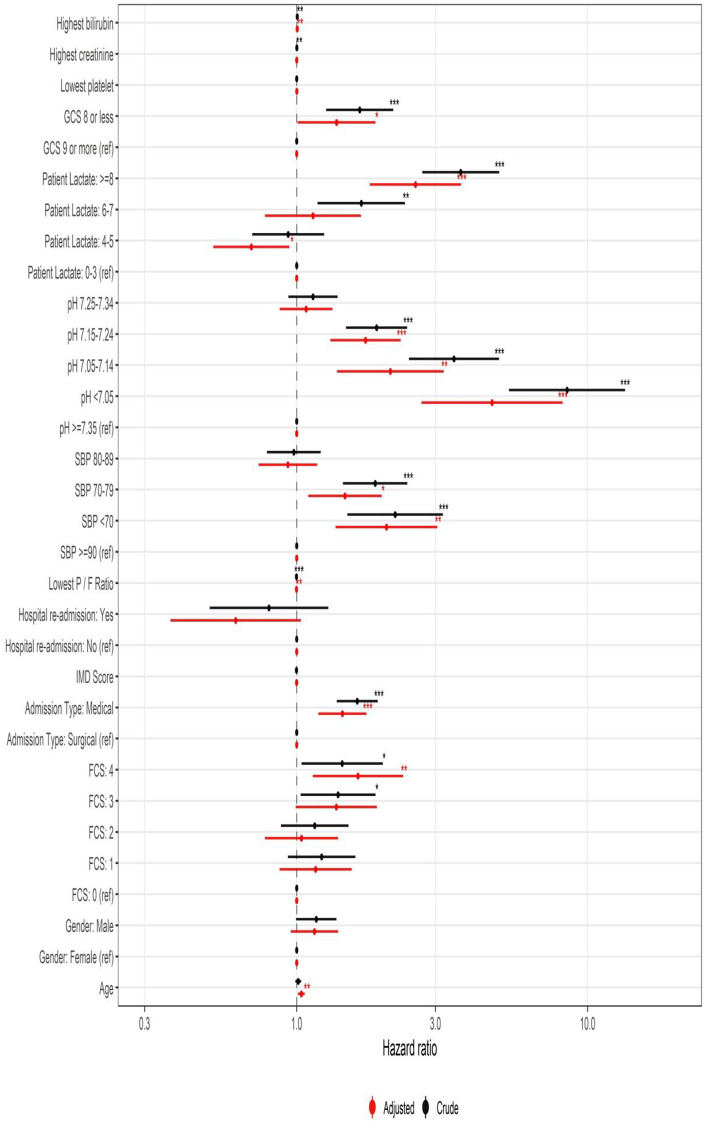
Adjusted and crude hazard ratios showing the effect of physiological variables and patient characteristics on survival.

Factors most associated with increased mortality were a medical diagnosis (hazard ratio (HR) 1.62 (95% CI 1.37–1.89), serum lactate concentration (6–8 mmol/l HR 1.67 (95% CI 1.18–2.36), > 8 mmol/ HR 3.66 (95% CI 2.70–4.97)), plasma pH (pH 7.15–7.25 HR 1.88 (95% CI 1.48–2.40), pH 7.05–7.15 HR 3.47 (95% CI 2.43–4.96), pH < 7.05 HR 8.5 (95% CI 5.37–13.45)), lower SBP (< 80mmHg HR 1.86 (95% CI 1.44–2.40), < 70 mmHg HR 2.18 (95% CI 1.49–3.18) and GCS ≤8 (HR 1.65 (95% CI 1.26–2.14). There were also significant differences for serum bilirubin (HR 1.004 (95% CI 1.001–1.007), serum creatinine (HR 1.001 (95% CI 1.000–1.001) and worsening P_a_O_2_/F_i_O_2_ ratio (HR 0.998 (95% CI 0.997–0.999) (Fig 3 and Table 3). A FCS of 3 or more was also associated with a worse outcome (FCS 3 HR 1.39 (95% CI 1.03–1.87), FCS ≥4 HR 1.43 (95% CI 1.04–1.98) (Fig 3 and S4 Table). There was no apparent effect of ICU readmission or socioeconomic deprivation on outcome (Fig 3 and S4 Table).

**Table 3:** Multivariable flexible parametric survival model using admission physiological variables for patients aged 80+ admitted to ICU as an emergency.

Variable	Multivariable analysis		
	Hazard ratio	P value	95% Confidence Interval
**Age**	1.04	0.007	1.01–1.07
**Male**	1.15	0.142	0.95–1.39
**FCS**			
**1**	1.16	0.303	0.87–1.55
**2**	1.04	0.799	0.78–1.39
**3**	1.36	0.056	0.99–1.89
**4**	1.62	0.008	1.14–2.32
**Medical**	1.44	<0.001	1.19–1.74
**IMD score**	0.999	0.702	0.995–1.003
**Readmission**	0.62	0.067	0.37–1.03
**P**_**a**_**O**_**2**_**/F**_**i**_**O**_**2**_ **ratio**	0.998	0.002	0.997–0.999
**Lowest Systolic BP**			
**<70mmHg**	2.04	0.001	1.36–3.05
**<80mmHg**	1.47	0.010	1.10–1.96
**<90mmHg**	0.93	0.560	0.74–1.18
**Lowest pH**			
**<7.05**	4.70	<0.001	2.67–8.21
**7.05–7.15**	2.10	0.001	1.38–3.20
**7.15–7.25**	1.72	<0.001	1.31–2.28
**7.25–7.35**	1.08	0.484	0.87–1.33
**Serum lactate**			
**4-6mmol/L**	0.70	0.020	0.52–0.94
**6-8mmol/L**	1.14	0.507	0.78–1.66
**>8mmol/L**	2.56	<0.001	1.79–3.67
**GCS ≤8**	1.37	0.045	1.001–1.865
**Lowest platelet count**	1.001	0.057	0.999–1.002
**Highest creatinine**	1.000	0.681	0.999–1.001
**Highest bilirubin**	1.004	0.007	1.001–1.007

Systolic blood pressure, pH and lactate classified as categorical variables to construct the prediction model

**FCS**: Functional comorbidity Score

**GCS**: Glasgow Coma Score

### Multivariable survival analysis

All *a priori* selected univariable variables were entered into the multivariable model. Independent predictors of outcome on multi-variable analysis were age (adjusted HR [aHR] 1.04 (95% CI 1.01–1.07), medical: surgical diagnosis (aHR 1.44 95% CI 1.19–1.74), lowest pH (pH 7.15–7.25 aHR 1.72 (95% CI 1.3.1–2.28), pH 7.05–7.15 aHR 2.10 (95% CI 1.38–3.20), pH < 7.05 aHR 4.70 (95% CI 2.67–8.21)), lowest SBP (SBP < 80mmHg aHR 1.47 (95% CI 1.10–1.96), SBP < 70 mmHg aHR 2.04 (95% CI 1.36–3.05)), serum lactate (> 8mmol/l aHR 2.56 (95% CI 1.79–3.67)), P_a_O_2_/F_i_O_2_ ratio (aHR 0.998 (95% CI 0.997–0.999)), GCS ≤8 (aHR 1.37 (95% CI 1.001–1.865), serum bilirubin (aHR 1.004 (95% CI 1.001–1.007)) and a FCS of ≥4 (aHR 1.62 (95% CI 1.14–2.32)) (Table 3). There was no contribution to outcome from socioeconomic deprivation, ICU readmission, gender or serum creatinine (Fig 3).

### Survival predictions based on multivariable survival model

For relatively minor degrees of physiological derangement (SBP 80–89 mmHg, pH > 7.35 and serum lactate 0–3 mmol/l) relative survival at one year and five years is 0.544 (95% CI 0.34–0.709) and 0.247 (0.084–0.454) respectively. For moderate physiological changes (SBP 70–79 mmHg, pH 7.15–7.24 and serum lactate 4–5 mmol/l) these figures fall to 0.316 (0.101–0.561) and 0.071 (0.005–0.267) respectively. For severe physiological derangement (pH < 7.05 and serum lactate > 8 mmol/), there was no survival to one and five regardless of SBP (S5 Table and S2 Fig). For patients with the most severe physiological derangements three (13.6%) survived to hospital discharge with a pH < 7.05, 12 (34%) survived with a SBP < 70mmHg and ten (20%) survived with a serum lactate > 8mmol/L (S6 Table). Median length of ICU stay in these categories was 1.1 days (interquartile range (IQR) 1.0–2.8) with a pH <7.05, 2.7 days (IQR 1.0–5.7) with a SBP <70mmHg and 1.5 days (IQR 1.0–5.6) with a serum lactate in excess of 8.0 mmol/L (S7 Table). Overall, 19 patients had all variables within the worst two physiological strata (pH< 7.15, SBP < 80mmHg and serum lactate > 6mmol/L). All of these patients died within the ICU with a median time to death of one day (range 0–22 days).

To assist clinical decision making we constructed a nomogram to predict probability of survival at annual intervals up to five years using Cox regression (S2 Fig). For example, based on our cohort data, an 80-year-old female post-surgical admission whose lowest SBP was > 90mmHg, with a pH of 7.34, a serum lactate of 1.5 mmol/l and a GCS > 8 could be expected to have one and five year survivals of > 80% and > 60% respectively. By contrast, an 85-year-old male medical patient with lowest SBP < 70mmHg, with a pH of 7.15, a serum lactate of 6.0 mmol/l and a GCS ≤ 8 will have an estimated survival at one and five years of 40% and 10% respectively.

## Discussion

Our study demonstrates that acute physiological derangement is the most important determinant of five-year survival in patients aged 80 and above following critical care admission. The most severe abnormalities of serum lactate and plasma pH resulted in no long–term survival. The impact of ICU admission on mortality appears to be prolonged, with relative survival in medical patients not returning to the baseline of the non-ICU population for the duration of the study. However, this is also likely to be related to a greater burden of co-morbid disease in medical patients. These findings need to be validated in multi-centre studies prior to widespread adoption.

Multiple previous studies have reported outcomes among elderly ICU patients [13, 26–32] with some incorporating all elderly admissions with others including only emergency [32] or medical patients [26] (S8 Table). Much of the published literature on elderly outcomes after ICU admission focuses on mortality up to one year after an episode of acute illness (S8 Table) with a paucity of longer-term outcome data. The only previous study reporting outcomes to five years included both elective and emergency patients and reports similar outcomes to our cohort at all time points up to five years [31]. Most authors have outlined the importance of acute physiological derangement to predict early mortality of elderly ICU patients [13, 26, 27, 31, 32]. The role of acute physiology beyond one year is less clear, with some reports of on-going effect of illness severity [26, 31, 32], some suggesting a limited effect [29] and others that functional status [30] and frailty [28, 33] are more important for long time frames.

Previous work has characterised the acute physiological derangement of patients and attempted to predict outcome on the basis of aggregated scoring systems that perform poorly in the elderly [12]. There has been no previous work describing outcomes up to five years associated with disaggregated components of these scores. We have shown the pronounced effect of serum lactate, plasma pH and SBP on survival for this age group with limited aHRs from other variables (Table 3 and Fig 3). In following patients for five years, we have also demonstrated, in common with earlier work [26, 31], that the effect of the ICU admission on mortality appears to extend beyond the acute admission. In particular, mortality amongst medical patients remains in excess of that of an age matched population for the duration of the study, an observation not previously described.

We have conducted a large single centre retrospective review of elderly outcomes with minimal loss of data during follow up and have presented comprehensive survival data for up to five years after ICU admission. We have incorporated data that are readily available at, or shortly after, ICU admission to assist in prognostication. In addition to illness severity data we have incorporated assessments of functional capacity and disease burden using the FCS. We have also explored the role that socioeconomic deprivation plays in the outcome of critically ill elderly patients, a factor that has not been explored previously in this population.

There are however, limitations to our study. Firstly, we have conducted a single centre observational study with potential for inherent selection biases. All critically ill patients in our centre are reviewed and triaged pre-admission as to the appropriateness of overly burdensome treatment. The survival experience of older patients will be strongly linked to the selection effect of ICU admission decision-making as well as case-mix. Given decision making and case-mix is likely to vary between centres, it is possible that the results from our study may not be sufficiently generalisable for wider use. Secondly, we currently do not routinely record the level of frailty in our patients. Given the importance of this as an outcome measure, we have used the FCS as a surrogate marker for incapacity. Thirdly, we have no information regarding the functional outcome of those surviving their episode of critical illness. Fourthly, our findings require validation in multi-centre studies.

The principal determinants of outcome in our study were those of circulatory shock. Even for those elderly patients with modest derangements of pH, serum lactate and SBP (SBP 70–79 mmHg, pH 7.15–7.2 and serum lactate 4–5 mmol/l) approximately 70% will not survive to one year. Based on these findings clinicians should prioritise their assessment of the shocked elderly patient to address the features of circulatory failure with judicious use fluid resuscitation, vasopressor and inotropic support as well as rapid source control for underlying infection. Additionally, these findings may also be used as a basis to initiate discussions with elderly patients and their families about potential benefits, harm and likely outcome of what may be burdensome and ultimately futile treatment. This may be particularly prudent in medical patients with multiple co-morbidities (FCS ≥ 4).

The median ICU length of stay for the whole cohort was short, at three days, reducing to one day for the most severely ill. This may indicate that the resource burden imposed by elderly patients upon the ICU may be less than expected. However, this potential low resource burden takes no account of the impact of overly burdensome care upon elderly patients and their families as alluded to above. A low impact on available resource should not be used to justify ICU admission in a scenario where futility is likely. All such management decisions should be made in the patient’s best interests allowing compassionate end of life care and symptom control where indicated rather than ICU admission.

We have demonstrated an overall five-year survival of 27% for elderly patients admitted to the ICU as an emergency. The corresponding figure for medical and surgical patients is 19% and 35% respectively. The presence of circulatory shock predicted particularly poor short and long term survival in elderly ICU patients. For those patients with a medical diagnosis and multiple co-morbidities frank discussion around patient wishes, best interests and likely outcomes may be appropriate. However, ultimately these findings will need to be validated in multi-centre studies prior to widespread implementation.

## Supporting information

S1 Fig Five year survival of medical and surgical patients compared to reference populations. The red and black lines represent modelled cumulative survival of the UK reference population (red line) and a local population (black line) with a socioeconomic profile the same as the study population.
(TIF)

S2 Fig Prediction model based on categorical data for survival up to five years for patients over the age of 80 years. (TIF)

S1 Table Proportional-hazards assumption tests. Table demonstrates output from the “estat phtest” command in STATA to test proportional hazards (PH)-assumptions for admission variables in a Cox-regression survival analysis model. The variables shown violate PH-assumptions. Therefore, a flexible parametric survival model was constructed for the survival analysis.
(DOCX)

S2 Table Flexible parametric survival model fitting. Table demonstrates Akaike’s Information Criteria (AIC) and Bayesian Information Criteria (BIC) values for the univariate variables that violated PH assumptions and the multivariate model for flexible parametric survival model fitting. We selected models with five internal splines based on lowest AIC and BIC values. d(f): degrees of freedom within stmp2 code (e.g. “stpm2 Lowest_pH, df(**5**) scale(hazard) eform”). The “estat ic” command was used to generate AIC and BIC values.
(DOCX)

S3 Table Mortality, cumulative survival and relative survival ratios at given time points up to five years from ICU admission. (DOCX)

S4 Table Univariable flexible parametric survival model using admission physiological variables for patients aged 80+ admitted to ICU as an emergency. Systolic blood pressure, pH and lactate classified as categorical variables to construct the prediction model.
(DOCX)

S5 Table Relative survival at given time points according to physiological categories. (DOCX)

S6 Table Outcomes at hospital discharge for systolic blood pressure, serum lactate and lowest pH recorded within the first twenty four hours following ICU admission. (DOCX)

S7 Table Median length of ICU stay for given physiological variables. (DOCX)

S8 Table Mortality at time points reported across previous studies of elderly patients admitted to ICU. (DOCX)

## References

[1] UK population by ethnicity: Age groups. https://www.ethnicity-facts-figures.service.gov.uk/british-population/demographics/age-groups/latest

[2] Haas LE, Karakus A, Holman R, Cihangir S, Reidinga AC, de Keizer NF. Trends in hospital and intensive care admissions in the Netherlands attributable to the very elderly in an ageing population. Crit Care. 2015;19:353 10.1186/s13054-015-1061-z 26423744 PMC4588268

[3] Nielsson MS, Christiansen CF, Johansen MB, Rasmussen BS, Tønnesen E, Nørgaard M. Mortality in elderly ICU patients: a cohort study. Acta Anaesthesiol Scand. 2014;58(1):19–26. 10.1111/aas.12211 24117049

[4] Fuchs L, Chronaki CE, Park S, Novack V, Baumfeld Y, Scott D, et al ICU admission characteristics and mortality rates among elderly and very elderly patients. Intensive Care Med. 2012;38(10):1654–61. 10.1007/s00134-012-2629-6 22797350 PMC5718912

[5] Jones A, Toft-Petersen AP, Shankar-Hari M, Harrison DA, Rowan KM. Demographic Shifts, Case Mix, Activity, and Outcome for Elderly Patients Admitted to Adult General ICUs in the United Kingdom, Wales, and Northern Ireland. Crit Care Med. 2020. 10.1097/CCM.0000000000004211 32205592

[6] Sprung CL, Artigas A, Kesecioglu J, Pezzi A, Wiis J, Pirracchio R, et al The Eldicus prospective, observational study of triage decision making in European intensive care units. Part II: intensive care benefit for the elderly. Crit Care Med. 2012;40(1):132–8. 10.1097/CCM.0b013e318232d6b0 22001580

[7] Boumendil A, Aegerter P, Guidet B, Network C-R. Treatment intensity and outcome of patients aged 80 and older in intensive care units: a multicenter matched-cohort study. J Am Geriatr Soc. 2005;53(1):88–93. 10.1111/j.1532-5415.2005.53016.x 15667382

[8] Guidet B, Leblanc G, Simon T, Woimant M, Quenot JP, Ganansia O, et al Effect of Systematic Intensive Care Unit Triage on Long-term Mortality Among Critically Ill Elderly Patients in France: A Randomized Clinical Trial. JAMA. 2017;318(15):1450–9. 10.1001/jama.2017.13889 28973065 PMC5710364

[9] Heyland DK, Stelfox HT, Garland A, Cook D, Dodek P, Kutsogiannis J, et al Predicting Performance Status 1 Year After Critical Illness in Patients 80 Years or Older: Development of a Multivariable Clinical Prediction Model. Crit Care Med. 2016;44(9):1718–26. 10.1097/CCM.0000000000001762 27075141

[10] Pandharipande PP, Girard TD, Jackson JC, Morandi A, Thompson JL, Pun BT, et al Long-term cognitive impairment after critical illness. N Engl J Med. 2013;369(14):1306–16. 10.1056/NEJMoa1301372 24088092 PMC3922401

[11] Anderson F, Downing GM, Hill J, Casorso L, Lerch N. Palliative performance scale (PPS): a new tool. J Palliat Care. 1996;12(1):5–11. 8857241

[12] Flaatten H, de Lange DW, Artigas A, Bin D, Moreno R, Christensen S, et al The status of intensive care medicine research and a future agenda for very old patients in the ICU. Intensive Care Med. 2017;43(9):1319–28. 10.1007/s00134-017-4718-z 28238055

[13] Bagshaw SM, Webb SA, Delaney A, George C, Pilcher D, Hart GK, et al Very old patients admitted to intensive care in Australia and New Zealand: a multi-centre cohort analysis. Crit Care. 2009;13(2):R45 10.1186/cc7768 19335921 PMC2689489

[14] de Rooij SE, Abu-Hanna A, Levi M, de Jonge E. Identification of high-risk subgroups in very elderly intensive care unit patients. Crit Care. 2007;11(2):R33 10.1186/cc5716 17346348 PMC2206449

[15] Guidet B, Hodgson E, Feldman C, Paruk F, Lipman J, Koh Y, et al The Durban World Congress Ethics Round Table Conference Report: II. Withholding or withdrawing of treatment in elderly patients admitted to the intensive care unit. J Crit Care. 2014;29(6):896–901. 10.1016/j.jcrc.2014.08.004 25216948

[16] Chin-Yee N, D’Egidio G, Thavorn K, Heyland D, Kyeremanteng K. Cost analysis of the very elderly admitted to intensive care units. Crit Care. 2017;21(1):109 10.1186/s13054-017-1689-y 28506243 PMC5433056

[17] Philippart F, Vesin A, Bruel C, Kpodji A, Durand-Gasselin B, Garçon P, et al The ETHICA study (part I): elderly’s thoughts about intensive care unit admission for life-sustaining treatments. Intensive Care Med. 2013;39(9):1565–73. 10.1007/s00134-013-2976-y 23765236

[18] Heyland DK, Barwich D, Pichora D, Dodek P, Lamontagne F, You JJ, et al Failure to engage hospitalized elderly patients and their families in advance care planning. JAMA Intern Med. 2013;173(9):778–87. 10.1001/jamainternmed.2013.180 23545563

[19] UK Government English Indices of Deprivation 2015. https://www.gov.uk/government/statistics/english-indices-of-deprivation-2015

[20] Groll DL, Heyland DK, Caeser M, Wright JG. Assessment of long-term physical function in acute respiratory distress syndrome (ARDS) patients: comparison of the Charlson Comorbidity Index and the Functional Comorbidity Index. Am J Phys Med Rehabil. 2006;85(7):574–81. 10.1097/01.phm.0000223220.91914.61 16788388

[21] UK Office of National Statistics Life Tables. https://www.ons.gov.uk/peoplepopulationandcommunity/birthsdeathsandmarriages/lifeexpectancies/datasets/nationallifetablesunitedkingdomreferencetables.

[22] Dickman PW, Coviello E. Estimating and modeling relative survival. The Stata Journal. 2015;15(1):186–215.

[23] Lambert P, Royston P. Further development of flexible parametric models for survival analysis. Stata J. 2009;9:265–90.

[24] Du X, Li M, Zhu P, Wang J, Hou L, Li J, et al Comparison of the flexible parametric survival model and Cox model in estimating Markov transition probabilities using real-world data. PLoS One. 2018;13(8):e0200807 10.1371/journal.pone.0200807 30133454 PMC6104919

[25] Dempsey, Ged, 2020, "Elderly ICU outcomes", Harvard Dataverse V2 10.7910/DVN/VNVJMS,

[26] Roch A, Wiramus S, Pauly V, Forel JM, Guervilly C, Gainnier M, et al Long-term outcome in medical patients aged 80 or over following admission to an intensive care unit. Crit Care. 2011;15(1):R36 10.1186/cc9984 21261976 PMC3222073

[27] de Rooij SE, Govers A, Korevaar JC, Abu-Hanna A, Levi M, de Jonge E. Short-term and long-term mortality in very elderly patients admitted to an intensive care unit. Intensive Care Med. 2006;32(7):1039–44. 10.1007/s00134-006-0171-0 16791666

[28] Flaatten H, De Lange DW, Morandi A, Andersen FH, Artigas A, Bertolini G, et al The impact of frailty on ICU and 30-day mortality and the level of care in very elderly patients (≥ 80 years). Intensive Care Med. 2017;43(12):1820–8. 10.1007/s00134-017-4940-8 28936626

[29] Atramont A, Lindecker-Cournil V, Rudant J, Tajahmady A, Drewniak N, Fouard A, et al Association of Age With Short-term and Long-term Mortality Among Patients Discharged From Intensive Care Units in France. JAMA Netw Open. 2019;2(5):e193215 10.1001/jamanetworkopen.2019.3215 31074809 PMC6512465

[30] Pietiläinen L, Hästbacka J, Bäcklund M, Parviainen I, Pettilä V, Reinikainen M. Premorbid functional status as a predictor of 1-year mortality and functional status in intensive care patients aged 80 years or older. Intensive Care Med. 2018;44(8):1221–9. 10.1007/s00134-018-5273-y 29968013

[31] Andersen FH, Flaatten H, Klepstad P, Romild U, Kvåle R. Long-term survival and quality of life after intensive care for patients 80 years of age or older. Ann Intensive Care. 2015;5(1):53. 10.1186/s13613-015-0053-0 PMC4456598 26055187

[32] Lown DJ, Knott J, Rechnitzer T, Maclsaac C. Predicting short-term and long-term mortality in elderly emergency patients admitted for intensive care. Crit Care Resusc. 2013;15(1):49–55. 23432502

[33] Muscedere J, Waters B, Varambally A, Bagshaw SM, Boyd JG, Maslove D, et al The impact of frailty on intensive care unit outcomes: a systematic review and meta-analysis. Intensive Care Med. 2017;43(8):1105–22. 10.1007/s00134-017-4867-0 28676896 PMC5501903

