# Supplementary figures and images for: Long term outcomes for elderly patients after emergency intensive care admission: A cohort study
Source: PLoS One. 2020 Oct 29;15(10):e0241244. doi: 10.1371/journal.pone.0241244 (PMC7595304; doi:10.1371/journal.pone.0241244)

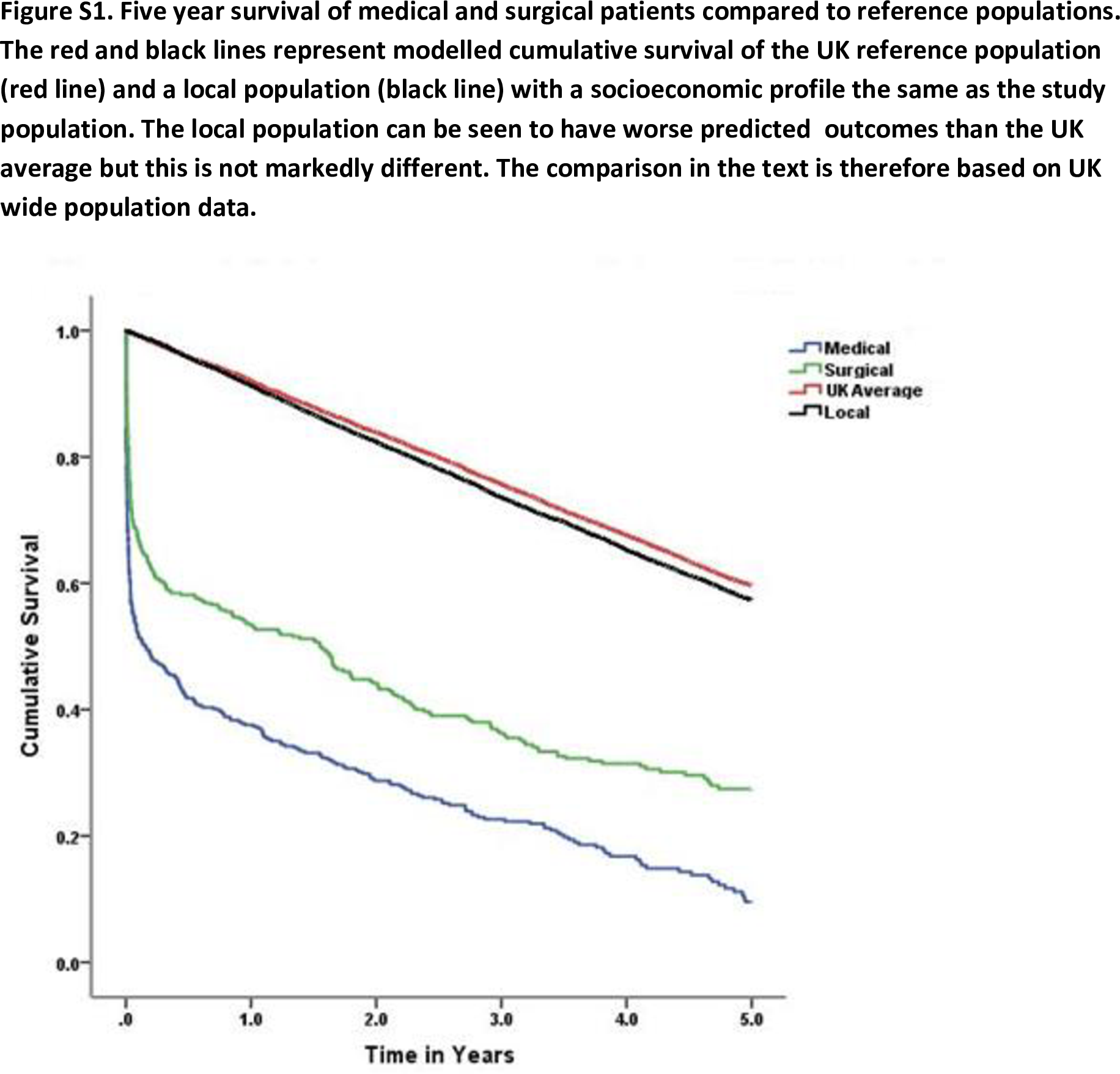

Supplement: S1 Fig — The red and black lines represent modelled cumulative survival of the UK reference population (red line) and a local population (black line) with a socioeconomic profile the same as the study population. (TIF) [file pone.0241244.s001.tif]

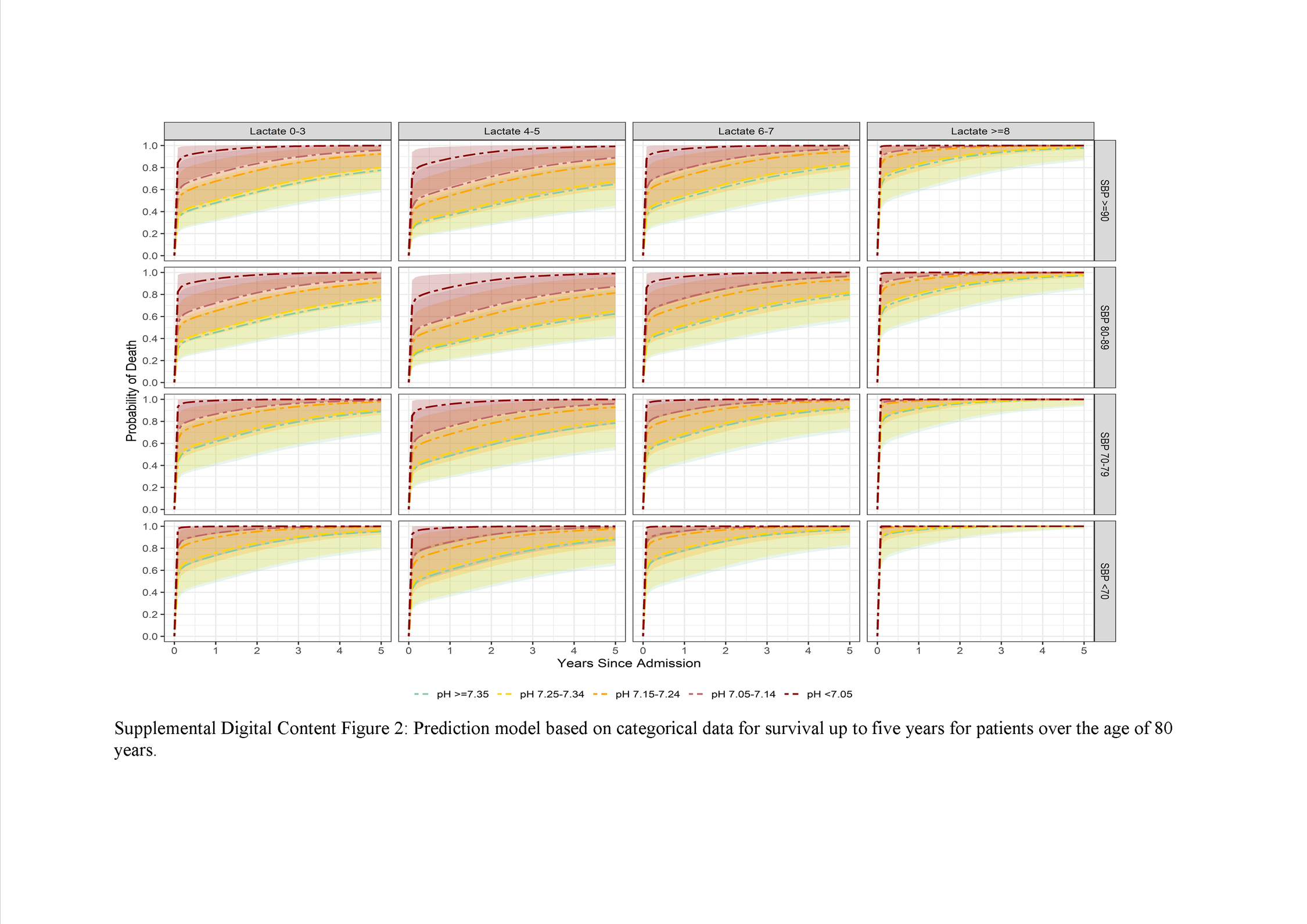

Supplement: S2 Fig — (TIF) [file pone.0241244.s002.tif]
