# Supplementary material for: Long term outcomes for elderly patients after emergency intensive care admission: A cohort study
Source: PLoS One. 2020 Oct 29;15(10):e0241244. doi: 10.1371/journal.pone.0241244 (PMC7595304; doi:10.1371/journal.pone.0241244)
Supplement: S1 Table — Table demonstrates output from the “estat phtest” command in STATA to test proportional hazards (PH)-assumptions for admission variables in a Cox-regression survival analysis model. The variables shown violate PH-assumptions. Therefore, a flexible parametric survival model was constructed for the survival analysis. (DOCX) [file pone.0241244.s003.docx]

**S1 Table:** **Proportional-Hazards Assumption Tests.** Table demonstrates output from the “estat phtest” command in STATA to test proportional hazards (PH)-assumptions for admission variables in a Cox-regression survival analysis model. The variables shown violate PH-assumptions. Therefore, a flexible parametric survival model was constructed for the survival analysis.

| Variable | Rho | Chi2 | df | P value |
| --- | --- | --- | --- | --- |
| Male | 0.11273 | 7.73 | 1 | 0.005 |
| Lowest Systolic BP | 0.08826 | 5.86 | 1 | 0.016 |
| P_a_O_2_/F_i_O_2_ ratio | 0.08053 | 4.06 | 1 | 0.044 |
| Lowest pH | 0.12714 | 10.37 | 1 | 0.001 |
| Lactate | -0.15572 | 14.31 | 1 | <0.001 |
