# Supplementary material for: Long term outcomes for elderly patients after emergency intensive care admission: A cohort study
Source: PLoS One. 2020 Oct 29;15(10):e0241244. doi: 10.1371/journal.pone.0241244 (PMC7595304; doi:10.1371/journal.pone.0241244)
Supplement: S2 Table — Table demonstrates Akaike’s Information Criteria (AIC) and Bayesian Information Criteria (BIC) values for the univariate variables that violated PH assumptions and the multivariate model for flexible parametric survival model fitting. We selected models with five internal splines based on lowest AIC and BIC values. d(f): degrees of freedom within stmp2 code (e.g. “stpm2 Lowest_pH, df(5) scale(hazard) eform”). The “estat ic” command was used to generate AIC and BIC values. (DOCX) [file pone.0241244.s004.docx]

**Table S2:** Flexible parametric survival model fitting. Table demonstrates Akaike's Information Criteria (AIC) and Bayesian Information Criteria (BIC) values for the univariate variables that violated PH assumptions and the multivariate model for flexible parametric survival model fitting. We selected models with five internal splines based on lowest AIC and BIC values. d(f): degrees of freedom within stmp2 code (e.g. “stpm2 Lowest_pH, df(**5**) scale(hazard) eform”). The “estat ic” command was used to generate AIC and BIC values

| Variable | df | AIC | BIC |
| --- | --- | --- | --- |
| Gender | 3  4  5 | 3410.402  3383.894  **3353.807** | 3433.979  3412.187  **3386.814** |
| Lowest Systolic BP | 3  4  5 | 3401.18  3374.783  **3344.915** | 3424.751  3403.068  **3377.915** |
| P_a_O_2_/F_i_O_2_ ratio | 3  4  5 | 3122.88  3092.81  **3062.713** | 3145.994  3120.546  **3095.072** |
| Lowest pH | 3  4  5 | 3067.686  3037.486  **3008.092** | 3090.813  3065.239  **3040.469** |
| Lactate | 3  4  5 | 2965.236  2942.138  **2916.305** | 2988.174  2969.663  **2948.418** |
| Multivariable model | 3  4  5 | 2833.211  2809.364  **2780.895** | 2928.993  2909.707  **2885.799** |
