# Supplementary material for: Long term outcomes for elderly patients after emergency intensive care admission: A cohort study
Source: PLoS One. 2020 Oct 29;15(10):e0241244. doi: 10.1371/journal.pone.0241244 (PMC7595304; doi:10.1371/journal.pone.0241244)
Supplement: S3 Table — (DOCX) [file pone.0241244.s005.docx]

**Table S3.** Mortality, cumulative survival and relative survival ratios at given time points up to five years from ICU admission

| **Time Point** | **Overall n=828** | | **Medical patients n= 423** | | | **Surgical patients n=405** | | |
| --- | --- | --- | --- | --- | --- | --- | --- | --- |
|  | **Mortality at time point n (%)** | **Cumulative survival n (%)** |  |  |  |  |  |  |
|  |  |  | **Mortality at time point n (%)** | **Cumulative survival n (%)** | **Relative survival ratio (95% CI)** | **Mortality at time point n (%)** | **Cumulative survival n (%)** | **Relative survival ratio (95% CI)** |
| 30 days | 324 (39) | 504 (61) | 197 (47) | 226 (53) | 0.536 (0.487 - 0.583) | 127 (31) | 278 (69) | 0.694 (0.646 - 0.737) |
| 90 days | 55(7) | 449 (54) | 23(5) | 203 (48) | 0.490 (0.440 - 0.538) | 32 (8) | 246 (61) | 0.623 (0.573 - 0.670) |
| 6 months | 33 (4) | 416 (50) | 23 (5) | 180 (43) | 0.441 (0.391 - 0.490) | 10 (2) | 236 (58) | 0.612 (0.560 - 0.660) |
| 1 year | 36 (4) | 380 (46) | 18 (4) | 162 (38) | 0.414 (0.363 - 0.464) | 18 (4) | 218 (54) | 0.587 (0.532 - 0.640) |
| 2 years | 62(7) | 318 (38) | 32 (8) | 130 (31) | 0.347 (0.295 - 0.401) | 30 (7) | 188 (46) | 0.541 (0.479 - 0.602) |
| 3 years | 45(5) | 273 (33) | 21 (5) | 109 (26) | 0.303 (0.249 - 0.361) | 24 (6) | 164 (40) | 0.498 (0.429 - 0.568) |
| 4 years | 28(3) | 245 (30) | 15 (4) | 94 (22) | 0.252 (0.195 - 0.316) | 13 (3) | 151 (37) | 0.494 (0.417 - 0.573) |
| 5 years | 22(27) | 223 (27) | 14 (3) | 80 (19) | 0.143 (0.085 - 0.220) | 8 (2) | 143 (35) | 0.501 (0.411 - 0.595) |
