# Supplementary material for: Long term outcomes for elderly patients after emergency intensive care admission: A cohort study
Source: PLoS One. 2020 Oct 29;15(10):e0241244. doi: 10.1371/journal.pone.0241244 (PMC7595304; doi:10.1371/journal.pone.0241244)
Supplement: S4 Table — Systolic blood pressure, pH and lactate classified as categorical variables to construct the prediction model. (DOCX) [file pone.0241244.s006.docx]

**Table S4:** Univariable flexible parametric survival model using admission physiological variables for patients aged 80+ admitted to ICU as an emergency. Systolic blood pressure, pH and lactate classified as categorical variables to construct the prediction model

| **Variable** | **Univariable analysis** | | |
| --- | --- | --- | --- |
|  | **Hazard ratio** | **P value** | **95% Confidence Interval** |
| **Age** | 1.01 | 0.301 | 0.99 – 1.04 |
| **Male** | 1.17 | 0.058 | 0.99 – 1.37 |
| **FCS:**  **1**  **2**  **3**  **4** | 1.22  1.15  1.39  1.43 | 0.147  0.295  0.030  0.028 | 0.93 – 1.59  0.88 – 1.51  1.03 – 1.87  1.04 – 1.98 |
| **Medical patient** | 1.62 | <0.001 | 1.37 – 1.89 |
| **IMD score** | 0.998 | 0.385 | 0.995 – 1.002 |
| **ICU Readmission** | 0.80 | 0.361 | 0.50 – 1.28 |
| **P_a_O_2_/F_i_O_2_ ratio** | 0.998 | <0.001 | 0.997 – 0.999 |
| **Lowest Systolic BP**  **<70mmHg**  **<80mmHg**  **<90mmHg** | 2.18  1.86  0.98 | <0.001  <0.001  0.833 | 1.49 – 3.18  1.44 – 2.40  0.79 – 1.21 |
| **Lowest pH**  **<7.05**  **7.05 – 7.15**  **7.15 – 7.25**  **7.25 – 7.35** | 8.50  3.47  1.88  1.14 | <0.001  <0.001  <0.001  0.196 | 5.37 – 13.45  2.43 – 4.96  1.48 – 2.40  0.94 – 1.38 |
| **Lactate**  **4-6mmol/L**  **6-8mmol/L**  **>8mmol/L** | 0.94  1.67  3.66 | 0.642  0.004  <0.001 | 0.70 – 1.24  1.18 – 2.36  2.70 – 4.97 |
| **GCS ≤8** | 1.65 | <0.001 | 1.26 – 2.14 |
| **Lowest platelet count (10*9/L)** | 0.999 | 0.929 | 0.999 – 1.001 |
| **Highest creatinine (µmol/L)** | 1.001 | 0.001 | 1.000 – 1.001 |
| **Highest bilirubin (µmol/L)** | 1.004 | 0.002 | 1.001 – 1.007 |
