# Supplementary material for: Long term outcomes for elderly patients after emergency intensive care admission: A cohort study
Source: PLoS One. 2020 Oct 29;15(10):e0241244. doi: 10.1371/journal.pone.0241244 (PMC7595304; doi:10.1371/journal.pone.0241244)
Supplement: S5 Table — (DOCX) [file pone.0241244.s007.docx]

**Table S5**. Relative survival at given time points according to physiological categories

| **SBP category** | **pH Category** | **Lactate Category** | **Relative survival at time period (95% CI)** | | | | |
| --- | --- | --- | --- | --- | --- | --- | --- |
|  |  |  | **One year** | **Two years** | **Three years** | **Four years** | **Five years** |
| > 90mmHg | ≥ 7.35 | 0-3 mmol/l | \| 0.521 (0.318-0.689) \| \| --- \| | 0.423 (0.221-0.611) | 0.338 (0.15-0.539) | 0.273 (0.103-0.477) | 0.223 (0.072-0.426) |
| > 90mmHg | 7.15-7.24 | 4-5 mmol/l | \| 0.456 (0.224-0.661) \| \| --- \| | 0.354 (0.139-0.579) | 0.271 (0.084-0.503) | 0.21 (0.051-0.44) | 0.165 (0.032-0.388) |
| > 90mmHg | < 7.05 | > 8 mmol/l | \| 0 (0-0.027) \| \| --- \| | 0 (0-0.009) | 0 (0-0.003) | 0 (0-0.001) | 0 (0-0) |
| 80-89 mmHg | ≥ 7.35 | 0-3 mmol/l | \| 0.544 (0.34-0.709) \| \| --- \| | 0.448 (0.242-0.635) | 0.364 (0.168-0.564) | 0.298 (0.118-0.504) | 0.247 (0.084-0.454) |
| 80-89 mmHg | 7.15-7.24 | 4-5 mmol/l | \| 0.48 (0.247-0.681) \| \| --- \| | 0.38 (0.158-0.602) | 0.296 (0.098-0.528) | 0.233 (0.062-0.466) | 0.186 (0.04-0.415) |
| 80-89 mmHg | < 7.05 | > 8 mmol/l | \| 0.001 (0-0.035) \| \| --- \| | 0 (0-0.012) | 0 (0-0.004) | 0 (0-0.001) | 0 (0-0) |
| 70-79 mmHg | ≥ 7.35 | 0-3 mmol/l | \| 0.384 (0.164-0.603) \| \| --- \| | 0.283 (0.092-0.513) | 0.204 (0.05-0.432) | 0.149 (0.027-0.366) | 0.111 (0.015-0.314) |
| 70-79 mmHg | 7.15-7.24 | 4-5 mmol/l | \| 0.316 (0.101-0.561) \| \| --- \| | 0.219 (0.048-0.466) | 0.148 (0.022-0.384) | 0.101 (0.01-0.318) | 0.071 (0.005-0.267) |
| 70-79 mmHg | < 7.05 | > 8 mmol/l | \| 0 (0-0.006) \| \| --- \| | 0 (0-0.001) | 0 (0-0) | 0 (0-0) | 0 (0-0) |
| < 70mmHg | ≥ 7.35 | 0-3 mmol/l | \| 0.265 (0.072-0.511) \| \| --- \| | 0.173 (0.031-0.412) | 0.11 (0.013-0.328) | 0.071 (0.005-0.264) | 0.047 (0.002-0.215) |
| < 70mmHg | 7.15-7.24 | 4-5 mmol/l | \| 0.202 (0.035-0.467) \| \| --- \| | 0.121 (0.012-0.366) | 0.07 (0.004-0.283) | 0.042 (0.001-0.222) | 0.025 (0-0.176) |
| < 70mmHg | < 7.05 | > 8 mmol/l | \| 0 (0-0.001) \| \| --- \| | 0 (0-0) | 0 (0-0) | 0 (0-0) | 0 (0-0) |
