# Supplementary material for: Long term outcomes for elderly patients after emergency intensive care admission: A cohort study
Source: PLoS One. 2020 Oct 29;15(10):e0241244. doi: 10.1371/journal.pone.0241244 (PMC7595304; doi:10.1371/journal.pone.0241244)
Supplement: S6 Table — (DOCX) [file pone.0241244.s008.docx]

**Table S6. Outcomes at hospital discharge for systolic blood pressure, serum lactate and lowest pH recorded within the first twenty four hours following ICU admission.**

| **Outcome at hospital discharge** | **Lowest systolic blood pressure in mmHg** | | | | |
| --- | --- | --- | --- | --- | --- |
|  | **< 70** | **< 80** | **<90** | **>90** | **Total** |
| Alive n (%) | 12 (34) | 37 (46) | 105 (66) | 353 (64) | 507 |
| Dead n (%) | 23 (66) | 44 (54) | 53 (34) | 200 (36) | 320 |
| Total | 35 | 81 | 158 | 553 | 827 |

| **Outcome at hospital discharge** | **Highest serum lactate in mmol/L** | | | | |
| --- | --- | --- | --- | --- | --- |
|  | **0-4** | **4-6** | **6-8** | **8** | **Total** |
| Alive n (%) | 359 (65) | 55 (64) | 16 (37) | 10 (20) | 440 |
| Dead n (%) | 190 (35) | 31 (36) | 27 (63) | 40 (80) | 288 |
| Total | 549 | 86 | 43 | 50 | 728 |

| **Outcome at hospital discharge** | **Lowest recorded pH** | | | | | |
| --- | --- | --- | --- | --- | --- | --- |
|  | **<7.05** | **7.05-7.15** | **7.15-7.25** | **7.25-7.35** | **>7.35** | **Total** |
| Alive n (%) | 3 (14) | 8 (21) | 45 (38) | 179 (64) | 215 (72) | 450 |
| Dead n (%) | 19 (86) | 31 (79) | 74 (62) | 100 (36) | 83 (28) | 307 |
| Total | 22 | 39 | 119 | 279 | 298 | 757 |
