# Supplementary material for: Long term outcomes for elderly patients after emergency intensive care admission: A cohort study
Source: PLoS One. 2020 Oct 29;15(10):e0241244. doi: 10.1371/journal.pone.0241244 (PMC7595304; doi:10.1371/journal.pone.0241244)
Supplement: S7 Table — (DOCX) [file pone.0241244.s009.docx]

**Table S7. Median length of ICU stay for given physiological variables.**

|  | **Median length of ICU stay in days (IQR)** |
| --- | --- |
| pH < 7.05 (n=22) | 1.06 (1.0-2.8) |
| pH 7.05-7.15 (n=39) | 2.4 (1.0-4.4) |
| pH 7.15-7.25 (n=119) | 4.0 (1.6-8.0) |
| pH 7.25-7.35 (n=279) | 3.8 (2.0-7.6) |
| pH > 7.35 (n=298) | 3.0 (1.9-5.7) |
| Systolic blood pressure < 70mmHg (n=35) | 2.67 (1.0-5.7) |
| Systolic blood pressure 70-80mmHg (n=81) | 3.0 (1.2-6.0) |
| Systolic blood pressure 80-90mmHg (n=158) | 4.0 (2.0-7.8) |
| Systolic blood pressure >90mmHg (n=553) | 3.0 (1.8-5.8) |
| Serum lactate 0-4mmol/L (n=549) | 3.6 (2.0-6.8) |
| Serum lactate 4-6mmol/L (n=86) | 4.0 (2.3-8.8) |
| Serum lactate 6-8mmol/L (n=43) | 3.1 (1.0-6.8) |
| Serum lactate > 8mmol/L (n=50) | 1.51 (1.0-5.6) |
