# Supplementary material for: Long term outcomes for elderly patients after emergency intensive care admission: A cohort study
Source: PLoS One. 2020 Oct 29;15(10):e0241244. doi: 10.1371/journal.pone.0241244 (PMC7595304; doi:10.1371/journal.pone.0241244)
Supplement: S8 Table — (DOCX) [file pone.0241244.s010.docx]

**Table S8**. Mortality at time points reported across previous studies of elderly patients admitted to ICU.

| **Study author** | **Year of publication** | **Patient cohort** | **Planned admissions (%)** | **Mortality at time point %** | | | | |
| --- | --- | --- | --- | --- | --- | --- | --- | --- |
|  |  |  |  | **30 day / hospital discharge** | **1 year** | **2 years** | **3 years** | **5 years** |
| Dempsey | - | Emergency | 0 | 39 | 54 | 62 | 67 | 73 |
| de Rooij (28) | 2007 | All admissions | 57 | 34 | 44 | - | - | - |
| Roch (27) | 2011 | Medical | 0 | 55 | 72 | 79 | - | - |
| Flatten (29) | 2017 | All admissions | 18 | 33 | - | - | - | - |
| Atramont (30) | 2019 | All admissions | Not reported | 30.5 | - | - | 61.4 | - |
| Pietilainen (31) | 2018 | All admissions | 26.5 | - | 38 | - | - | - |
| Bagshaw (13) | 2009 | All admissions | 39 | 24 | - | - | - | - |
| Andersen (32) | 2015 | All admissions | 12.7 | 40.5 | 58 | 63.4 | 68.8 | 77.8 |
| Lown (33) | 2013 | Emergency | 0 | - | 46.3 | - | - | - |
